# Long Non-Coding RNAs, Novel Offenders or Guardians in Multiple Sclerosis: A Scoping Review

**DOI:** 10.3389/fimmu.2021.774002

**Published:** 2021-12-07

**Authors:** Abbas Jalaiei, Mohammad Reza Asadi, Hani Sabaie, Hossein Dehghani, Jalal Gharesouran, Bashdar Mahmud Hussen, Mohammad Taheri, Soudeh Ghafouri-Fard, Maryam Rezazadeh

**Affiliations:** ^1^ Molecular Medicine Research Center, Tabriz University of Medical Sciences, Tabriz, Iran; ^2^ Department of Medical Genetics, Faculty of Medicine, Tabriz University of Medical Sciences, Tabriz, Iran; ^3^ Department of Molecular Medicine, School of Medicine, Birjand University of Medical Sciences, Birjand, Iran; ^4^ Department Pharmacognosy, College of Pharmacy, Hawler Medical University, Erbil, Iraq; ^5^ Skull Base Research Center, Loghman Hakim Hospital, Shahid Beheshti University of Medical Sciences, Tehran, Iran; ^6^ Institute of Human Genetics, Jena University Hospital, Jena, Germany; ^7^ Department of Medical Genetics, School of Medicine, Shahid Beheshti University of Medical Sciences, Tehran, Iran

**Keywords:** lncRNAs, multiple sclerosis, neurodegenerative disease, polymorphism, expression

## Abstract

Multiple sclerosis (MS), a chronic inflammatory demyelinating disease of the central nervous system, is one of the most common neurodegenerative diseases worldwide. MS results in serious neurological dysfunctions and disability. Disturbances in coding and non-coding genes are key components leading to neurodegeneration along with environmental factors. Long non-coding RNAs (lncRNAs) are long molecules in cells that take part in the regulation of gene expression. Several studies have confirmed the role of lncRNAs in neurodegenerative diseases such as MS. In the current study, we performed a systematic analysis of the role of lncRNAs in this disorder. In total, 53 studies were recognized as eligible for this systematic review. Of the listed lncRNAs, 52 lncRNAs were upregulated, 37 lncRNAs were downregulated, and 11 lncRNAs had no significant expression difference in MS patients compared with controls. We also summarized some of the mechanisms of lncRNA functions in MS. The emerging role of lncRNAs in neurodegenerative diseases suggests that their dysregulation could trigger neuronal death *via* still unexplored RNA-based regulatory mechanisms. Evaluation of their diagnostic significance and therapeutic potential could help in the design of novel treatments for MS.

## Introduction

Multiple sclerosis (MS) is a chronic inflammatory demyelinating disease of the central nervous system (CNS) and one of the most common neurodegenerative diseases worldwide (1). Pathogenic mechanisms underlying MS development have not been determined up to now. Clinically, different MS subtypes have been identified, including relapsing–remitting (RR), secondary progressive (SP), and primary progressive (PP) subtypes. These subtypes are heterogeneous among affected individuals in terms of clinical course as well as genetic background (2). Complex interactions between genetic susceptibility and environmental factors lead to this neurodegenerative disease. Both innate and adaptive immune-mediated inflammatory mechanisms contribute to the demyelination and neurodegeneration in the context of MS. Previous studies have demonstrated that the inflammatory immune cells such as CD4 T-helper cells (Th1 and Th17) are the main contributors in disease pathogenesis (3, 4). The presence of these cells in the CNS is associated with neuronal demyelination, which can subsequently result in neuroinflammation and neurodegeneration (5, 6). Th17 cells that produce IL-17 are regarded as important inflammatory effectors in this disorder (7). However, the impact of Th17 cells in the pathogenesis of MS is not entirely dependent on the production of this cytokine, and it is supposed that an array of inflammatory factors is responsible in this regard (8). For example, expression of high amounts of the C-C chemokine receptor 6 (CCR6) on the cell surface of Th17 cells (9) facilitates the entry of these cells into the CNS *via* the choroid plexus (10). Th17 cells also participate in the pathoetiology of MS through production of other proinflammatory cytokines including TNF-α (11).

In recent years, genome-wide association studies (GWAS) and genetic mapping have nominated several candidate loci and variants in autoimmune conditions. However, MS pathogenesis cannot be explained by the genetic susceptibility factors alone. A large amount of evidence has revealed that long non-coding RNAs (lncRNAs) have critical roles in the regulation of cellular immunological pathways and autoimmunity. This new class of non-coding RNA (ncRNAs) contains a large part of the transcriptional output in the human genome but low protein-coding potential (12).

In the current review, we focus on recent reports performed on the roles of lncRNAs in MS pathogenesis. Then, we illustrate the role of some specific lncRNAs and their target genes. Therefore, our manuscript provides new insights into understanding the molecular etiology, diagnosis, and management of MS.

### Long Non-Coding RNA Classification and Function

LncRNAs are a class of ncRNAs with sizes more than 200 nt and no protein-coding potential. They are commonly transcribed by RNA Pol II (13). LncRNAs have been detected in a variety of species such as animals, plants, and prokaryotes. The majority of them have a 5′ cap structure, multiple exons, and 3′ polyadenylated tails and are spliced in a way similar to mRNAs (14). Since lncRNAs do not encode proteins, they used to be called as “dark matter.” However, recent studies have demonstrated that they are regulatory molecules and play important roles in several biological processes (14, 15), including gene expression at the epigenetic, transcriptional, and posttranscriptional levels. The vital mechanisms of epigenetic regulation consist of DNA methylation, histone modification, and ncRNA-mediated regulation. Emerging evidence revealed that the normal execution of biological events is controlled by a combination of ncRNAs and transcription factor (TF)-mediated epigenetic modifications (16). Studies on the role of lncRNAs suggest that their dysregulation could trigger neuronal death *via* still unexplored RNA-based regulatory mechanisms (17). Gene signature in human CNS is precisely regulated by several mechanisms. LncRNAs have a substantial impact on normal neural development, so their abnormal expression affects development and progression of neurodegenerative diseases (18).

According to databases such as the NONCODE (version v5.0) (19), the number of lncRNAs in human has been estimated to be higher than the number of protein-coding genes. The classification of lncRNAs is based on subcellular localization, function, interaction with the protein-coding gene, their size, and their association with protein-encoding genes. Based on their association with protein-encoding genes, they can be categorized to different classes such as sense, intergenic, bidirectional, intronic, antisense, and divergent lncRNAs (20, 21). Long intergenic non-coding RNA (lincRNA) genes are an important group of ncRNAs that participate in many biological processes, such as regulation of gene expression. They also play an essential role in many autoimmune and inflammatory diseases (22). In the current study, we performed a systematic analysis of the role of lncRNAs in MS.

## Methods

Review question: Which lncRNAs have been dysregulated in multiple sclerosis?

### Inclusion/Exclusion Criteria

The inclusion criteria were as follows: 1) original studies, 2) studies focusing on the expression of lncRNAs in MS patients, 3) studies that confirmed results by RT-PCR, 4) studies with a sample of blood or tissue of human or animal model, and 5) studies that evaluated polymorphisms on lncRNAs. The following documents were excluded from this study: letters, reviews, *in vitro* studies, or papers with insufficient data.

### Search Strategy

The current scoping review was performed according to the PRISMA statement (23). PubMed, Web of Science, ProQuest, and Scopus databases were searched to identify all published studies up to August 10, 2021.

### Study Selection

Following the abovementioned search method, all obtained papers were loaded into EndNote version 20. Then, duplicate studies were removed. The title and abstracts of the remaining studies were evaluated, and their full texts were screened using the inclusion criteria. Then, lncRNAs with a role in the pathogenesis of MS were included.

### Data Extraction

The required data were extracted using a self-constructed data extraction table. Author and year of publication, origin, sample type, studied patients, method for lncRNA analysis, identified lncRNAs and expression pattern, and polymorphisms were extracted from the studies.

**Figure 1:**
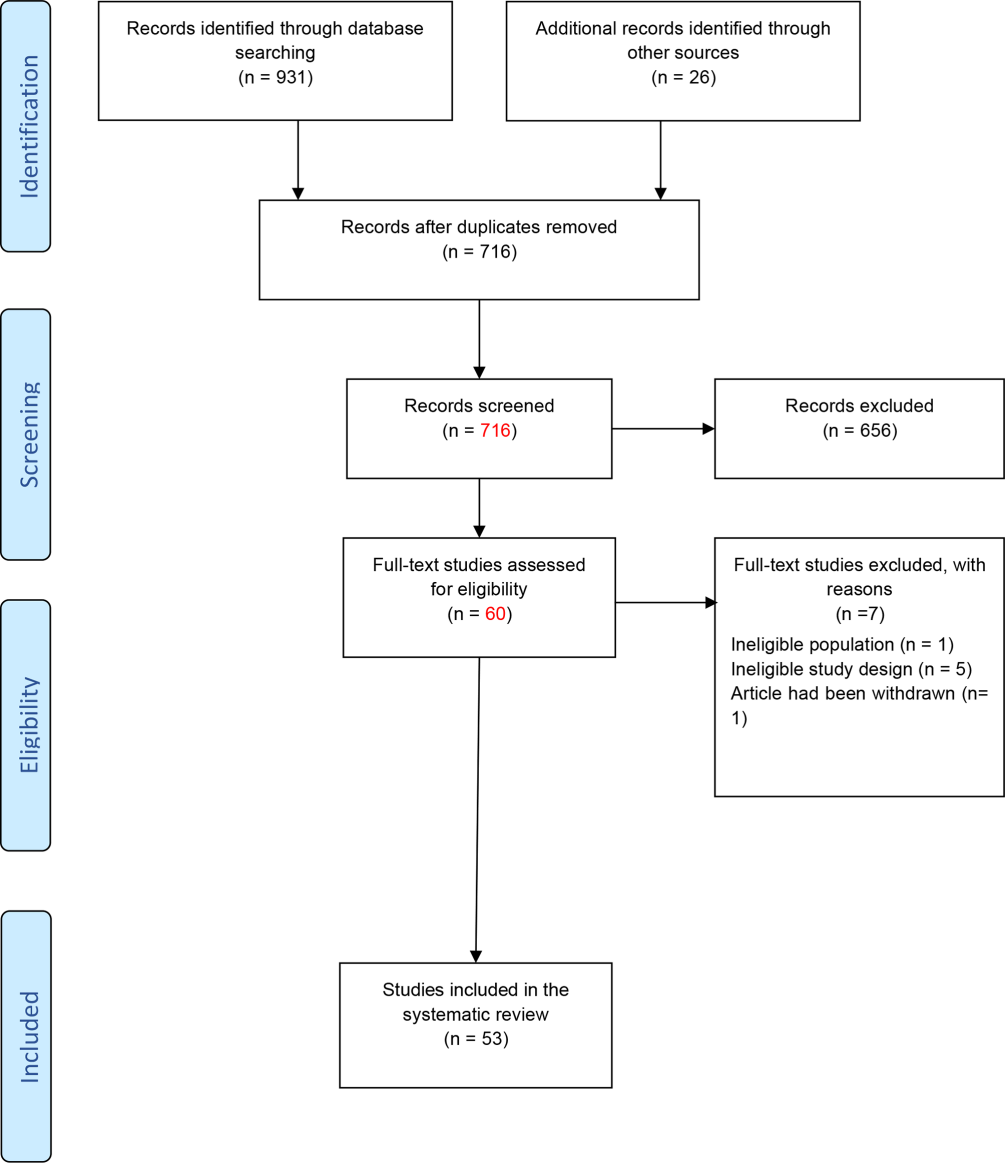
Flowchart of the study (23).

**Figure 1** shows the flowchart of the study.

## Results

As shown in **Figure 1**, a total of 931 studies were identified through searching PubMed, Web of Science, ProQuest, and Scopus databases, and 26 studies were identified from other databases. After removing duplicated articles, 716 studies remained. In the next step, based on the evaluation of titles and abstracts, 656 studies were excluded and 60 studies remained. The full text of the articles was evaluated based on our inclusion criteria. After evaluation of the full text, seven studies were removed due to lack of inclusion criteria. At last, 53 studies remained for our systematic review. Among the included studies, 47 studies were conducted on human samples (24–70), 7 studies used animal models (45, 71–76), and only 1 research was conducted on both human samples and animal model (45). Also, 33 studies were conducted in the Iranian population (24–26, 28–34, 36, 38–43, 45–55, 57, 58, 61, 66, 67), 9 studies were in China (68–76), 5 studies were in Egypt (37, 62–65), 4 studies were in Italy (27, 35, 59, 60), 1 study was in Russia (44), and 1 study was in the Netherlands (56). A total of 44 studies evaluated the expression of lncRNAs in MS patients (24, 26–31, 34–43, 45, 46, 48, 50–54, 56–61, 63–65, 67–76), while 9 other studies analyzed polymorphisms of lncRNAs (25, 32, 33, 44, 47, 49, 55, 62, 66). The details of the included studies are summarized in **Tables 1**, **2**.

**Table 1:** Details of the included human studies.

Author	Year	Origin	LncRNA measurement technique	Sample type	Number of studied patients	Identified lncRNA/expression pattern	Polymorphism	Ref
Bahrami et al.	2021	Iran	RT-PCR	PBMCs	50 RRMS	Lnc-DC ↑		(24)
					50 controls			
Bahrami et al.	2020	Iran	T-ARMS PCR	PBMCs	300 patients		TRPM2-AS1, rs933151 HNF1A-AS1, rs7953249	(25)
					300 controls			
Bina et al.	2017	Iran	RT- PCR	PBMCs	36 RRMS	Inc-IL-7R [NS]		(26)
					30 Controls			
Cardamone et al.	2019	Italy	Microarray assay validation by RT-PCR	PBMCs	190 cases	MALAT1 ↑		(27)
					182 controls			
Dastmalchi et al.	2018	Iran	RT-PCR	PBMCs	50 RRMS	NEAT1 ↑		(28)
					50 controls	TUG1 ↑		
						PANDA ↑		
Dastmalchi et al.	2018	Iran	TaqMan RT-PCR	PBMCs	50 RRMS	UCA1 ↑		(29)
					50 controls	CCAT2 ↑		
Dehghanzad et al.	2020	Iran	RT-PCR	PBMCs	39 MS	TOB1-AS1 ↑		(30)
					32 controls			
Eftekharian et al.	2019	Iran	T-ARMS-PCR Confirmed by the Sanger method	PBMCs	428 MS		MALAT1 rs619586, rs3200401	(32)
					505 controls			
Eftekharian et al.	2019	Iran	T-ARMS PCR	PBMCs	400 MS	GAS5 ↑	rs2067079	(33)
					410 controls		rs6790	
Eftekharian et al.	2019	Iran	TaqMan RT-PCR	PBMCs	50 RRMS	NNT-AS1 ↑		(34)
					50 controls			
Eftekharian et al.	2017	Iran	TaqMan RT-PCR	PBMCs	50 RRMS	THRIL ↑		(31)
					50 controls	FAS-AS1 ↓		
						PVT1 ↓		
Fenoglio et al.	2018	Italy–Belgium	Real-time PCR validated with TaqMan and lastly confirmed by droplet digital PCR	PBMCs	27 RRMS	MALAT1 ↓, MEG9 ↓, NRON ↓, ANRIL ↓, TUG1 ↓, XIST ↓, SOX2OT ↓, GOMAFU ↓, HULC ↓, BACE-1AS ↓		(35)
					13 PPMS			
					31 controls			
Ganji et al.	2019	Iran	RT-PCR	PBMCs	50 RRMS	GSTT1-AS1 ↓		(36)
					50 controls	IFNG-AS1 ↓		
Ghaiad et al.	2020	Egypt	RT-PCR	PBMCs	72 MS	APOA1-AS1 ↑		(37)
					28 controls	IFNG-AS1 ↑		
						RMRP ↑		
Gharesouran et al.	2019	Iran	TaqMan RT-PCR	PBMCs	50 RRMS	MALAT1 ↑		(39)
					50 controls	HOTAIRM1 ↑		
Gharesouran et al.	2019	Iran	TaqMan RT-PCR	PBMCs	50 RRMS	OIP5-AS1 ↓		(40)
					50 controls			
Gharesouran et al.	2018	Iran	TaqMan RT-PCR	PBMCs	50 RRMS	GAS5 ↑		(38)
					50 controls			
Gharzi et al.	2018	Iran	RT-PCR	PBMCs	50 RRMS	BDNF-AS1 [NS]		(41)
					50 controls			
Ghoveud et al.	2020	Iran	RT-PCR	PBMCs	50 RRMS	RP11-530C5.1 ↑		(42)
					25 controls	AL928742.12 ↓		
Hosseini et al.	2019	Iran	RT-PCR	PBMCs	50 RRMS	AC007278.2 ↑		(43)
					25 controls	IFNG-AS1-001 ↑		
						IFNG-AS1-003 ↑		
Kozin et al.	2020	Russia	PCR-RFLP performed by TaqMan RT-PCR	PBMCs	444 RRMS		PVT1	(44)
					96 SPMS		rs2114358	
					406 controls		rs4410871	
Masoumi et al.	2019	Iran	RT-PCR	Human brain tissue	5 RRMS	MALAT1 ↓		(45)
					5 controls			
Mazdeh et al.	2019	Iran	RT-PCR	PBMCs	50 RRMS	AFAP1-AS1 ↑		(46)
					50 controls			
Mazdeh et al.	2019	Iran	T-ARMS PCR	PBMCs	402 RRMS		LncRNA H19	(47)
					392 controls		rs2839698	
							rs217727	
Moradi et al.	2020	Iran	RT-PCR confirmed by RFLP	PBMCs	300 RRMS		GAS5, rs55829688 and NR3C1, rs6189/6190, rs56149945, rs41423247	(49)
					300 controls			
Moradi et al.	2019	Iran	RT-PCR	PBMCs	20 RRMS	NR003531.3(MEG3a) ↓		(48)
					10 controls	AC00061.2_201 [NS]		
						AC007182-6 [NS]		
Pahlevan Kakhki et al.	2019	Iran, North Khorasan, Sistani	RT-PCR	PBMCs	North Khorasan 30 MS, 30 controls	THRIL, North Khorasan ↑		(51)
					Sistani 21 MS, 21 controls	Sistani ↓		
						Inc-DC [NS] both groups		
Pahlevan Kakhki et al.	2018	Iran	RT-PCR	PBMCs	42 RRMS	HOTAIR ↑		(50)
					32 controls	ANRIL [NS]		
Patoughi et al.	2020	Iran	RT-PCR	PBMCs	50 RRMS	PINK1-AS ↑		(53)
					50 controls			
Patoughi et al.	2019	Iran	TaqMan RT-PCR	PBMCs	50 RRMS	GAS8-AS1 ↑		(52)
					50 controls			
Rahmani et al.	2020	Iran	RT-PCR	PBMCs	83 RRMS	RORC ↑		(54)
					44 controls	DDX5 ↑		
						RMRP ↑		
Rezazadeh et al.	2018	Iran	T-ARMS-PCR	PBMCs	410 RRMS		ANRIL, rs1333045, rs4977574, rs1333048, rs10757278	(55)
					419 controls			
Rodríguez-Lorenzo	2020	Netherlands	Ref-seq validated by RT-PCR	Brain tissue	6 MS patients	HIF1A-AS3 ↑		(56)
					6 controls			
Safa et al.	2020	Iran	RT-PCR	PBMCs	50 RRMS	LINC00305 ↓		(57)
					50 controls	lnc-MKI67IP-3 ↓		
						HNF1A-AS1↓		
						MIR31HG [NS]		
						NKILA [NS]		
						ADINR [NS]		
						CHAST [NS]		
						DICER1-AS1 [NS]		
Safa et al.	2020	Iran	RT-PCR	Venous blood	40 RRMS	SPRY4-IT1 ↓		(58)
					40 controls	HOXA-AS2 ↓		
						LINC-ROR ↓		
						MEG3 ↓		
Santoro et al.	2020	Italy	RT-PCR	Serum	16 SPMS, 12 PPMS	TUG1 ↑		(59)
					8 controls	LINC00293 ↑		
						RP11-29G8.3 ↑		
Santoro et al.	2016	Italy	RT-PCR	Serum	12 RRMS	NEAT1 ↑		(60)
					12 controls	TUG1 ↑		
						RN7SKRNA ↑		
Sayad et al.	2019	Iran	TaqMan RT-PCR	PBMCs	50 RRMS	HULC ↑		(61)
					50 controls			
Senousy et al.	2020	Egypt	TaqMan RT-PCR	Serum	108 RRMS	GAS5 ↑	rs2067079	(62)
					104 controls		rs1625579	
Shaker et al.	2021	Egypt	RT-PCR	PBMCs	74 RRMS, SPMS	LincR-Ccr2-5′AS ↓		(64)
					60 controls	THRIL ↑		
Shaker et al.	2019	Egypt	RT-PCR	PBMCs	42 RRMS	LincR-Gng2-5′ ↑		(63)
					18 SPMS	LincREpas1-3′as ↓		
					60 controls			
Shaker et al.	2019	Egypt	RT-PCR	Serum	45 RRMS	MALAT1 T ↑		(65)
					45 controls	Inc-DC ↑		
Taheri et al.	2020	Iran	T-ARMS-PCR	PBMCs	403 MS patients		HOTAIR, rs12826786, rs1899663, rs4759314	(66)
					420 controls			
Teimuri et al.	2019	Iran	RT-PCR	PBMCs	25 RRMS	AL450992.2 ↓		(67)
					25 SPMS	AC009948.5 ↓		
					25 controls	RP11-98D18.3 ↓		
						AC007182.6 ↓		
Zhang et al.	2018	China	Microarray assay validation by RT-PCR	PBMCs	36 RRMS	lncDDIT4 ↑		(69)
					26 controls			
Zhang et al.	2017	China	RT-PCR	PBMCs	34 RRMS	Linc-MAF4 ↑		(70)
					26 controls			
Zhang et al.	2016	China	RT-PCR	PBMCs	26 RRMS	MYO3B-AS1 (ENSG00000231898.3) ↑		(68)
					26 controls	AC104809.2 (ENSG00000233392.1) ↓		
						AC120045.1 (ENSG00000259906.1) ↓		
						LncRNA XLOC_010931 ↓		
						LncRNA XLOC_009626 ↑		
						LncRNA XLOC_010881 ↑		

RT-PCR, real-time PCR; T-ARMS-PCR, tetra-primer amplification refractory mutation system-PCR; PBMCs, peripheral blood mononuclear cells; RRMS, relapsing–remitting multiple sclerosis; SPMS, secondary progressive multiple sclerosis; upregulation, ↑; downregulation, ↓; NS, not significant; rs, reference SNP.

**Table 2:** Details of the included animal studies.

Author	Year	Origin	LncRNA measurement technique	Sample type	Type of EAE model	Identified lncRNA/expression pattern	Ref
Bian et al.	2020	China	Microarray assay validation by q-PCR	Spleen tissue	Not mentioned	GM15575 ↑	(71)
Duan et al.	2018	China	RT-PCR	Microglia	Cuprizone-induced demyelination	HOTAIR ↑	(72)
Guo et al.	2017	China	Microarray confirmed by RT-PCR	Spleen tissue	Myelin oligodendrocyte glycoprotein (MOG) peptide-induced EAE	1700040D17Rik ↓	(73)
Liu et al.	2021	China	RT-PCR	Spinal cords or astrocyte	MOG peptide-induced EAE	GM13568 ↑	(74)
Masoumi et al.	2019	Iran	RT-PCR	Lumbar spinal cord tissue	MOG peptide-induced EAE	MALAT1 ↓	(45)
Sun et al.	2017	China	Microarray assay validation by RT-PCR	Microglia	MOG peptide-induced EAE	GAS5 ↑	(75)
Yue et al.	2019	China	RT-PCR Western blot	Microglia BV2 cells	MOG peptide-induced EAE	TUG1 ↑	(76)

RT-PCR, real-time PCR; EAE, autoimmune encephalomyelitis; upregulation, ↑; downregulation, ↓.

**Figure 2:**
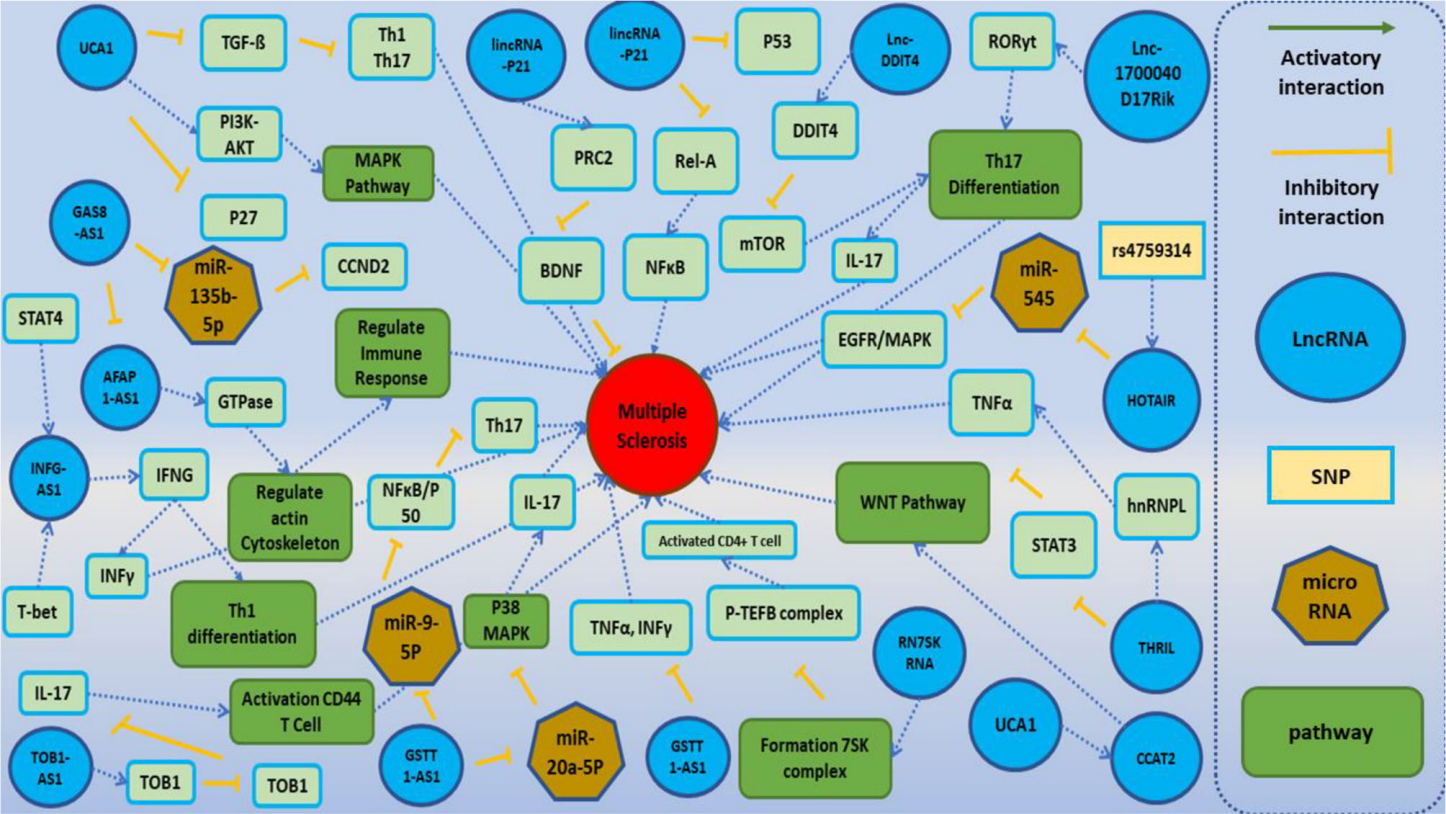
A schematic diagram of the role of several lncRNAs involved in the modulation of the main molecular cascades in multiple sclerosis (MS). One of the main pathophysiological mechanisms associated with the MS involves T cells subsets [regulatory T cells (Treg), Th1, Th2, and Th17 cells]. Dysregulation of these subsets activates inflammatory cascades and cytokine secretion and ultimately leads to demyelination within the brain and spinal cord and neuronal damage. Lnc-DC has been shown to be upregulated in PBMCs of MS patients. Upregulation of this lncRNA activates Toll-like receptor 4 (TLR4) and TLR9. TLR4 has a central role in the secretion of inflammatory cytokines such as IL-1, IL-6, and IL-17 and suppresses Treg cells. Also, TLR4 increases the differentiation of Th17 through inhibition of miR-30a (24, 65). Moreover, lnc-DDIT4 is upregulated in the PBMCs of MS patients. This lncRNA binds to DDIT4 and regulates immune response and differentiation of Th17 (69). BDNF-AS has a role in the recruitment of PRC2 and inhibition of the neuroprotective factor BDNF (41). GSTT1-AS1 inhibits the progression of MS through inhibition of secretion of IFN-γ and TNF-α (36). TUG1 activates p38 MAPK signaling pathway through suppression of miR-20a-5p, so downregulation of TUG1 decreases Th17 differentiation. UCA1 has a role in the regulation of activity of PI3K–AKT, ERK1/2, and MAPK cascades and Th17 differentiation. Also, this lncRNA has interaction with another lncRNA, namely, CCAT2. CCAT2 induces WNT cascade signaling and enhances the production of inflammatory cytokines (28, 59).

Recently, several studies revealed the involvement of lncRNAs in the pathogenesis of MS. **Figure 2** demonstrates the function of several lncRNAs that are involved in the pathogenesis of MS.

### The Role of LncRNAs in the Pathophysiology of MS

#### LncRNAs Participating in Adaptive Immune Response or Inflammation

Linc-MAF-4 and lnc-DDIT4 are two upregulated lncRNAs in MS patients which are involved in the regulation of immune responses and inflammation (69, 70). DDIT4 is a cytoplasmic protein that is upregulated during DNA damage. Also, it inhibits the mTORC1 pathway which is a crucial regulator of the immune response (77). Since the mTOR pathway causes differentiation of Th17 and subsequent production of IL-17, it can be a key pathogenic player in MS (78, 79). Lnc-DDIT4 directly binds to and increases DDIT4 expression; thus, it suppresses the differentiation of Th17 (69). Therefore, lnc-DDIT4 might directly regulate Th17 cell differentiation and contribute to the pathogenesis of MS. Linc-MAF-4 is a lincRNA located in the minus strand of 16q23.2, almost 150 kb apart from the gene encoding MAF (19). This lincRNA has an important role in regulating differentiation of Th1/2 cells. MAF is the Th2 lineage-specific TF facilitating Th2 differentiation (70). Linc-MAF-4 is a Th1 lineage-specific factor that recruits chromatin remodeling factors LSD1 and EZH2 to inhibit MAF transcription and elevate Th1 differentiation and IFN-γ production (15). So, linc-MAF-4 can contribute in the pathogenesis of MS. Another study has identified six lncRNAs with abnormal expression in MS. ENSG00000231898.3 (MYO3B-AS1), XLOC_009626, and XLOC_010881 were upregulated, while ENSG00000233392.1 (AC104809.2), ENSG00000259906.1 (AC120045.1), and XLOC_010931 showed downregulation (68).

##### LincR-Gng2-5′, LincR-Epas1-3′as, and LincR-Ccr2-5′AS

LincR-Gng2-5**′** and lincR-Epas1-3 loci were firstly identified by Hu et al. in Th1 and Th2 cells regulated by signal transducer and activator of transcription 4 (STAT4) and (STAT6), respectively (22). According to the data from lncRNAdb (80), LNCipedia (version 5.2), and Ensemble genome browser 99, LincR-Gng2-5′ is located on chromosome 14q22.1 on the plus strand and has a transcript size of 1,233 bp. LincR-Epas1-3**′**as is located on chromosome 2p.21 on the positive strand and has 758 bp length. They are located in an important place rich in genes with immune regulatory functions. Since they act as enhancers, they might participate in the regulation of neighboring genes, thus modulating immune responses (63). LincR-Gng2-5′ is upregulated in MS patients, while LincR-Epas1-3′as is downregulated in these patients. Dysregulation of these lncRNAs has a role in the pathoetiology of MS through affecting the balance between Th1 and Th2 cells (22, 81). LincR-Ccr2-5′AS is another lncRNA that is expressed in Th2 and has association with GATA-binding protein 3 (GATA3), the “master regulator” of Th2. Shaker et al. have reported the downregulation of lincR-Ccr2-5′AS in MS patients and the subsequent decrease in the production of Th2 cytokines (64).

##### GSTT1-AS1 and IFNG-AS1

Glutathione S-transferase, Theta1-Anti Sense1 (GSTT1-AS1), also known as lncRNA-CD244, is a novel 284-bp lncRNA, located on the minus strand 22q11.23 with partial overlap with 5′ UTR of the *GSTT1* gene (19, 82). This lncRNA was originally discovered as an lncRNA with a crucial role in the pathogenesis of tuberculosis (83). Ganji et al. show downregulation of GSTT1-AS1 in MS patients. Since this lncRNA suppresses the expression of TNF and INFG through recruitment of the epigenetic complex PRC2 and *via* the EZH2 enzyme complex, it might be involved in the pathogenesis of MS (36).

IFNG-AS1 has been firstly identified as a transcript with a possible role in the regulation of immune system function (84). Also known as Tmevpg1, it is a 1,791-bp intergenic lncRNA located on the plus strand on 12q15 (19), adjacent to the *INFG* gene (85). It has been shown to be dysregulated in several immune-related disorders (83, 86). This lincRNA acts as an important checkpoint for the expression of IFNG in Th1 cells (87).

##### AC007278.2 (Expression in T Cells)

Another lncRNA is a 1,200-bp intronic lncRNA, AC007278.2, also known as Lnc-IL18R1-1. This lncRNA is located on the plus strand of the 2q12.1 chromosome and has two exons (19). AC007278.2 has a specific expression in Th1 cells. It is located within the introns of the protein-coding genes *IL18RAP* and *IL18R1*, with important roles in Th1 cell differentiation (43). Several studies revealed significant correlations between IL18RAP and IL18R1 and their association with the lncRNA AC007278.2. On the other hand, elevated expression of IL18RAP and IL18R1 is involved in the differentiation of Th1 cells and the pathogenesis MS. During Th1 differentiation, STAT4 and IL-12 recruit chromatin remodeling complexes. Induction of histone acetylases and DNA methylases promotes the expression of IL18RAP and IL18R1 and the release of IL-18 and IL-12 which trigger the differentiation of Th1 and the release of pro-inflammatory cytokines and eventually the progression of MS (43, 88, 89).

##### TOB1-AS1

TOB1 antisense RNA 1 (TOB1-AS1) is transcribed from the opposite orientation of the *TOB1* gene on chromosome 17q21.33, a region with an important role in maintaining immune tolerance (19). Dehghanzad et al. demonstrated the abnormal expression levels of TOB1-AS1 and its targets genes *TOB1*, *TSG*, and *SKP2* in the blood of MS. Downregulation of TOB1-AS1 might cause dysregulation of the target genes and participate in the progression of MS (30). TOB1-AS1 enhances the expression of the *TOB1* gene *via* suppressing the production of IL-2 (90). An *in vitro* study revealed the positive feedback between TOB1 and S-phase kinase-associated protein 2 (SKP2). Elevation of TOB1-AS1 levels causes increased TOB1 and thus increased the TSG levels (30).

##### RMRP

Rahmani et al. demonstrated that RORC, DDX5, and RMRP have been significantly upregulated in patients with MS (54). RORC and DDX5 can affect MS pathogenesis through regulation of Th17 differentiation and the production of inflammatory cytokines such as IL-17A, IL-17F, and IL-22.

#### LncRNAs With Roles in Innate Immune Response

##### Lnc-DC and THRIL

TNF and HNRNPL-related immunoregulatory long non-coding RNA (THRIL) is a lincRNA located on the minus strand of the 12q24.31 chromosome. This lncRNA plays an important role in the regulation of the innate immune system (19). This lncRNA has been among the dysregulated lncRNAs in MS (31). THRIL regulates TNF-α expression *via* its interaction with heterogeneous nuclear ribonucleoprotein L (hnRNPL) and persuades a transcriptional-activating complex, finally connecting to the TNF-α promoter (91). THRIL can suppress STAT3 (51).

Lnc-DC (also known as Wfdc21) is a non-coding RNA gene on the minus strand of chromosome 17q23.1, which was firstly identified by Wang et al. to have an important role in the differentiation of dendritic cells and the regulation of the immune response (92, 93). Lnc-DC positively regulates STAT3 resulting in the differentiation of monocyte cell to dendritic cells (92). This lncRNA is involved in the pathogenesis of sepsis (93), coronary artery disease (94), pre-eclampsia (95), MS (51), and systemic lupus erythematosus (SLE) (96). Xie et al. showed the role of lnc-Dc on the regulation of TLR4 (93). Lnc-DC through the TLR9/STAT3 axis can regulate apoptosis and immune responses, thus can participate in the pathogenesis of MS (97, 98). Bahrami et al. demonstrated the upregulation of lnc-DC level in HLADRB1*15:01-negative MS patients compared with healthy controls (24).

#### LncRNAs Having a Role in Response to DNA Damage

##### LincRNA-p21 (Expression in T Cell)

P21-associated ncRNA DNA damage-activated (PANDA) is a lincRNA located on the minus strand 6p21.2. It has a role in response to DNA damage in a p53-dependent pathway (15). Dastmalchi et al. revealed the upregulation of this lncRNA in the peripheral blood of MS patients (28). PANDA controls the cell cycle through suppression of proapoptotic-related genes (15, 99). Dysregulation of the expression of this lncRNA in oligodendrocytes and neurons is associated with the release of free radicals and activation of the apoptosis process (100).

#### LncRNAs Involved in the Regulation of the Cell Cycle

##### TUG1, UCA1, and CCAT2

UCA1, CCAT2, and TUG1 are a subgroup of lncRNAs that have a role in the regulation of the cell cycle. UCA1 is located in the plus strand of chromosome 19p13.12 (19). It participates in the pathogenesis of several cancers such as colorectal, breast, and bladder cancer through increasing cell proliferation, apoptosis-resistant cells, invasion, and drug resistance induction (101). UCA1 *via* modulation of the PI3K–AKT, ERK1/2, and MAPK pathways can regulate the proliferation of cells in various cancers (102). Dastmalchi et al. revealed the upregulation of UCA1 in the blood of MS patients. This lncRNA *via* inhibiting cell cycle inhibitors such as p27 may cause increased proliferation of T cells (29).

CCAT2 is an intergenic lncRNA on the plus strand of the 8q24.21 chromosome (19). This lncRNA acts as an oncogene and participates in the metastasis, chromosomal instability, and tumor growth in colon cancer (103). Both UCA1 and CCAT2 can regulate the expression of genes participating in WNT pathway (104).

Fenoglio et al. showed the downregulation of TUG1 in MS patients compared with controls (35). TUG1 exerts a repressor function *via* recruitment of the PRC2 complex. Its promoter has many conserved binding sites for p53, thus after DNA damage, p53 regulates cell cycle and apoptosis *via* upregulation of TUG1 (35, 105, 106). TUG1 has been found to be upregulated in the serum and PBMCs of RRMS patients (28, 59, 60). TUG1 targets and suppresses different miRNAs such as miR-20a-5p, which has a role in the regulation of p38 MAPK signaling pathway. p38 MAPK promotes the production of proinflammatory cytokines. Downregulation of miR-20a-5p by TUG1 activates p38 MAPK signaling and MS progression (60).

The growth arrest-specific 5 (GAS5) has been recognized as a lncRNA with a possible role in normal growth arrest in T cells. This lncRNA plays a central role in the suppression of glucocorticoid receptor (GR). Gharesouran et al. revealed the correlation between GAS5 and nuclear receptor subfamily 3 group C member 1 (NR3C1) (38). Sun et al. demonstrated that GAS5 can inhibit the transcription factor IRF4, thus suppressing the generation of T cells (75).

#### LncRNAs With a Role in the CNS

##### GOMAFU

MIAT or GOMAFU is a lincRNA on the plus strand of 22q12.2 (19), which is highly expressed in the CNS and is suggested to have an important role in regulating the neural stem cell differentiation into oligodendrocytes (107). Fenoglio et al. showed the downregulation of this lncRNA in the blood of MS patients (35). GOMAFU using its repetitive sequence binds to the splicing factor 1 (SF1) protein and prevents the function of the spliceosome complex. Thus, deregulation of GOMAFU causes advent of alternative splicing patterns (108). GOMAFU has a possible role in inflammatory and neurodegenerative processes (35).

##### OIP5-AS1

OIP5-AS1 (Cyrano) was firstly detected in zebrafish models and it was suggested that it has a role in the development of the CNS (109). Kim et al. revealed that OIP5‐AS1 causes a reduction in the stability a cyclin G‐associated kinase (GAK) mRNA with important roles for mitotic progression (110). It seems that this lncRNA exerts its role in the suppression of cell proliferation through reducing GAK levels by associating with the RNA-binding proteins (RBPs) like HUR1 (ELAV-like protein 1). HuR1 is a protein that in humans is encoded by the ELAVL1 and is regarded as a member of the ELAVL proteins. HUR1 contains three RNA-binding domains and binds to cis-acting AU-rich elements. Since the *HuR1* gene is expressed in astrocytes, it might have a role in autoimmune diseases such as encephalomyelitis and MS (111).

##### BDNF-AS

Brain-derived neurotrophic factor-antisense RNA (BDNF-AS) is a 191-kb-long conserved lncRNA (112), located in the opposite orientation of BDNF on the 11p14.1. It negatively regulates the expression of BDNF at the mRNA and protein levels (113). BDNF is a neuroprotective factor that is synthesized in the brain and is expressed at a high level in the CNS. It has diverse functions such as the promotion of neuronal survival and elevation of growth, maturation, and synaptic plasticity. BDNF is produced and released by neurons and immune cells such as T and B cells under the circumstance of inflammation of the CNS in MS patients (114). BDNF-AS recruits PRC2 and inhibits BDNF expression (113).

#### Other LncRNAs

##### NEAT1

This lncRNA has been shown to be upregulated in MS patients compared with healthy individuals (59). NEAT1 plays an important role in the formation of paraspeckle, a nuclear body that comprises numerous protein factors. NEAT1 has been shown to be co-localized with splicing factor proline/glutamine-rich (SFPQ) and NonPOU domain containing, octamer-binding (NONO) (115). Also, NEAT1 is activated by the Toll-like receptor 3 (TLR3)–p38 pathway in antiviral response or endogenous agonists that bind to TLR3 (116, 117). Imamura et al. revealed that upregulation of NEAT1 causes activation and excess IL-8 production *via* enhancing the relocation of SFPQ proteins from the IL-8 promoter (118).

##### RN7SK RNA

The lincRNA 7SK small nuclear (RN7SK RNA) is transcribed from the plus strand of the 6p12.2 chromosome. It is involved in the formation of the 7SK snRNP complex with other specific proteins (HEXIM1/2, LARP7, and PIP7S) that can inhibit approximately half of the activity of the cellular kinase P-TEFb complex (119, 120). The P-TEFb complex and its protein component Cdk9/cyclin T1 heterodimer have a role in the activation of CD4+ T cells. So, upregulated RN7SK RNA may cause disturbance in the P-TEFb complex with resulting regulation effects on CD4+ T cells, thus participating in autoimmune diseases such as idiopathic inflammatory myopathy (IIM) and MS (59).

##### AFAP1-AS1

Actin Filament-Associated Protein 1 Antisense RNA 1 (AFAP1-AS1) is a conserved non-coding RNA transcribed from the plus strand of chromosome 4p16.1 on the opposite strand of the AFAP1 locus. This lncRNA regulates the expression of AFAP1 at the translation level (121). AFAP1**-**AS1 was found to modulate AFAP1 and act as an adapter molecule that links other proteins such as SRC and PKC with a hypothetical function in blood–brain barrier (BBB) integrity. BBB dysfunction in MS patients allows the enormous influx of immune cells into the brain and, after a series of interactions, leads to demyelination (122). Based on the bioinformatics analyses, AFAP1-AS1 affected the expression of molecules with a vital role in the actin cytoskeleton signaling pathway such as multiple small GTPase family members. As small GTPases are involved in the regulation of immunity and inflammation response, its dysregulation leads to disease progression in many diseases such as autoimmune diseases (123). Upregulation of AFAP1-AS1 promotes metastasis *via* modulation actin filament integrity (124). Due to its antiapoptotic properties in peripheral immune cells, it might be involved in the pathogenesis of MS (40).

##### GAS8-AS1

A previous study showed that GAS8-AS1 is a tumor suppressor and regulates the expression of another lncRNA, namely, AFAP1-AS1 (125). GAS8-AS1 has been downregulated, while AFAP-AS1 has been upregulated in MS patients. Regarding the role of AFAP1-AS1 in the pathogenesis and progression of MS, it can be hypothesized that dysregulation of GAS8-AS1 might be involved in the pathogenesis of MS (40, 125). Zha et al. revealed that GAS8-AS1 negatively regulated the expression of UCA1. UCA1 has been shown to regulate various signaling pathways such as FGFR1/ERK and TGF-β (126). TGF-β has a role in the inflammatory condition and acts as an anti-inflammatory factor to inhibit Th1 and Th17 cells (127), so upregulation of GAS8-AS1 resulting in the downregulation of UCA1 and reduced TGF-β might cause progression and aggregate MS.

##### PINK1-AS

PTEN-induced kinase 1-AS (PINK1-AS) is an intronic non-coding RNA transcribed from the minus strand of chromosome 1p36.12 on the opposite strand of the PINK1 locus. This lncRNA regulates the expression of PINK1. Patoughi et al. (53) revealed the upregulation of the expression level of the PINK1-AS in male MS patients compared with male healthy controls. This might be due to the existence of a gender-based regulatory direction for PINK1-AS expression or variance in the pathogenic process of disease in female and male MS patients. PINK1 is a serine/threonine kinase that preserves the mitochondria and supports its normal function (128). Further studies by Fenoglio et al. have identified 10 lncRNAs with abnormal expression. These lncRNAs consist of MALAT1, MEG9, NRON, ANRIL, TUG1, XIST, SOX2OT, GOMAFU, HULC, and BACE-1AS (35).

The highly upregulated liver cancer (HULC) is another lncRNA found to be upregulated in MS patients in one study (61), whereas Fenoglio et al. have reported an opposite result (35). This lncRNA attaches to miR-200a-3p and also acts as an endogenous sponge for miR-122. Since miR-122 has an anti-inflammatory effect and is significantly downregulated in the blood of MS patients, HULC may be involved in the progression of MS. On the other hand, HULC activates miR-200a-3p/ZEB1 signaling. miR-200a plays an important role in the regulation of the TLR4 pathway and ZEB1 has a neuroprotective protein (129).

#### Dysregulated LncRNAs in the Animal Model of MS

One of the useful animal models of MS is EAE mice that share several characteristics with MS. However, there are few studies in this area. Yue et al. (76) demonstrated the abnormal activity of the TUG1/miR-9-5p/NF-κB1/p50 axis in the mouse model of MS. In fact, upregulation of TUG1 causes suppression of miR-9-5p and an increase in the expression of NF-κB1/p50. This transcription factor causes activation of Th17 cell and the production of IL-17 and IL-6. NF-κB also regulates matrix metalloproteinases (MMPs). Downregulation of TUG1 leads to increased levels of miR-9-5p and a decrease in NF-κB1/p50.

Another study by Guo and colleagues showed that lncRNA-1700040D17Rik is a specific mouse lincRNA that is located adjacent to the *RORγt* gene on chromosome 3 and is downregulated in EAE (73). Then, an *in vitro* approach revealed that IL23R-CHR is a soluble IL23R that counteracts IL-23 and blocks its signaling pathway, thus inhibiting differentiation of Th17 cell (130). These findings demonstrated that 1700040D17Rik regulates the expression of RORγt, which is an essential transcription factor for Th17 (73).

Liu et al. revealed that IL-9 inducing lncRNA Gm13568 in astrocytes has interaction with CBP/P300. It promotes Notch1 pathway activation and is involved in the construction of inflammatory cytokines in astrocytes in the progression of EAE development (74).

#### Variants Within LncRNAs and Association With MS

According to the important roles of lncRNAs in the regulation of immune responses, it is expected that functional variants within their coding region or adjacent to them can affect the risk of MS. However, there are few studies on this issue. Bahrami et al. have evaluated the association between rs933151 and rs7953249 polymorphisms in TRPM2-AS and HNF1-AS1, respectively, and MS risk in the Iranian population. They revealed that rs7953249 within HNF1-AS1 has an association with C-reactive protein (CRP) (25).

Taheri et al. assessed the association between three SNPs (rs12826786, rs1899663, and rs4759314) within HOTAIR and MS in 403 Iranian MS patients and 420 controls. Their results showed that the G allele of rs4759314 might be involved in the risk of MS (66).

## Conclusion

In conclusion, the pathogenesis of MS is highly complex including several molecular signaling pathways. Most of the abovementioned studies have assessed the expression of lncRNAs in serum or PBMCs. Although several of these lncRNAs have essential roles in the CNS processes, modulation of peripheral immune responses is the most appreciated route of participation of lncRNAs in the pathogenesis of MS. Few studies have assessed the expressions of lncRNAs in the brain tissues of EAE models. An important study in this field has identified dysregulation of Gm14005, Gm12478, mouselincRNA1117, AK080435, and mouselincRNA0681 in brain tissues of affected animals. Notably, inflammation has been among the mostly enriched pathways among dysregulated genes (131). This observation further emphasized the importance of inflammation-related lncRNAs in the pathoetiology of MS.

In the current review, we highlighted the function of various lncRNAs in the MS pathway. Although few studies have addressed this issue, it is predicted that genomic variation within lncRNAs affecting their function or expression may contribute to the risk of MS or response of subjects to treatments. It has been determined that lncRNAs have roles in the development of the immune system and nerve cells. Further studies are required for understanding the mechanism of lncRNA involvement in the pathogenesis of MS.

## Author Contributions

AJ, MT, BH, and SG-F wrote the draft and revised it. MR, HS, JG, and MA collected the data and designed the figures. HD performed the bioinformatics analysis. All authors contributed to the article and approved the submitted version.

## Conflict of Interest

The authors declare that the research was conducted in the absence of any commercial or financial relationships that could be construed as a potential conflict of interest.

## Publisher’s Note

All claims expressed in this article are solely those of the authors and do not necessarily represent those of their affiliated organizations, or those of the publisher, the editors and the reviewers. Any product that may be evaluated in this article, or claim that may be made by its manufacturer, is not guaranteed or endorsed by the publisher.

## Glossary

lncRNA: long non-coding RNA
MS: multiple sclerosis
RT-PCR: real-time polymerase chain reaction
AFAP1-AS1: actin filament-associated protein 1 antisense RNA 1
RRMS: relapsing–remitting multiple sclerosis
SPMS: secondary progressive multiple sclerosis
CNS: central nervous system
HOTAIR: Hox transcript antisense intergenic RNA
miRNAs: microRNAs
CD4+ T cells: T helper cells
CD8+ T cells: cytotoxic T cells
GWAS: genome-wide association studies
BDNF: brain-derived neurotrophic factor
BDNF-AS: BDNF antisense RNA
NR3C1: nuclear receptor subfamily 3 group C member 1
PRC2: polycarbonate 2 suppressor complex
DDIT4: DNA-damage-inducible transcript 4
mTORC1: mammalian target of rapamycin complex 1
lncDDIT4: lncRNA DDIT4
Th17: T helper 17 cell
Tregs: regulatory T cells
IFN-γ: interferon gamma
hnRNPs: heterogeneous nuclear ribonucleoproteins
DC: dendritic cells
lnc-DC: lncRNA expressed in DC
PANDA: P21-associated ncRNA DNA damage-activated
FAS-AS1: FAS antisense transcript 1
linc-MAF-4: A lncRNA
THRIL: TNF-α and heterogeneous nuclear ribonucleoprotein L
PVT1: plasmacytoma variant translocation 1
GAK: cyclin G-associated kinase
HuR1: Huantigen R
SIRT1: silent information regulator 1
OIP5-AS1: OIP5 antisense RNA 1
TUG1: taurine-upregulated gene
IL-8: interleukin 8
SFPQ: splicing factor proline- and glutamine-rich
IL-17: interleukin 17
STAT4: *signal transducer and activator of transcription 4*
EZH2: enhancer of zeste homolog 2
TNF-α: tumor necrosis factor alpha
TLR4: Toll-like receptor 4
NF-κB: nuclear factor kappa-light-chain-enhancer of activated B cells
MAPK: mitogen-activated protein kinase
PI3K: phosphoinositide 3-kinases
ERK1/2: extracellular signal-regulated kinases 1/2
AKT: protein kinase B
WNT: Wnt signaling pathway
SF1: splicing factor 1
GAK: G-associated kinase
NonPOU: non-POU domain-containing octamer-binding protein
P-TEFb: positive transcription elongation factor
BBB: blood–brain barrier
FGFR1: fibroblast growth factor receptor 1
ERK: extracellular signal-regulated kinase
TGF-β: transforming growth factor beta
CRP: C-reactive protein
PINK1-AS: PTEN-induced kinase 1-AS
HIF1-AS3: hypoxia-inducible factor 1-AS3
RMRP: RNA component of the mitochondrial RNA-processing endoribonuclease (RNase MRP)
GATA3: GATA-binding protein 3
GR: glucocorticoid receptor
HULC: highly upregulated liver cancer
ZEB1: zinc finger and homeodomain transcription factor 1
